# Variable-angle high-angle annular dark-field imaging: application to three-dimensional dopant atom profiling

**DOI:** 10.1038/srep12419

**Published:** 2015-07-24

**Authors:** Jack Y. Zhang, Jinwoo Hwang, Brandon J. Isaac, Susanne Stemmer

**Affiliations:** 1 Materials Department, University of California, Santa Barbara, California 93106-5050, U. S. A.

## Abstract

Variable-angle high-angle annular dark-field (HAADF) imaging in scanning transmission electron microscopy is developed for precise and accurate determination of three-dimensional (3D) dopant atom configurations. Gd-doped SrTiO_3_ films containing Sr columns containing zero, one, or two Gd dopant atoms are imaged in HAADF mode using two different collection angles. Variable-angle HAADF significantly increases both the precision and accuracy of 3D dopant profiling. Using image simulations, it is shown that the combined information from the two detectors reduces the uncertainty in the dopant depth position measurement and can uniquely identify certain atomic configurations that are indistinguishable with a single detector setting. Additional advances and applications are discussed.

High-angle annular dark-field (HAADF) imaging in scanning transmission electron microscopy (STEM) has become a key analysis technique in materials and nanosciences because it provides intuitively interpretable, atomic-resolution images that are sensitive to the atomic number (Z). For example, the *Z*-number sensitivity allows for the detection of individual impurity atoms inside crystals^1^,^2^,^3^,^4^,^5^,^6^,^7^,^8^,^9^. The key idea behind the development of HAADF-STEM is *angular selection of the scattered signal*: the detector’s annular geometry collects only the electrons scattered to large angles^10^. This avoids collecting the Bragg reflections and allows for images that are not subject to contrast reversals, at the expense of signal. Recent developments in STEM-based signal detection have advanced this basic concept to gain additional information. For example, it has long been known that the *Z*-number dependence of the signal varies with the scattering angle^10^,^11^. Annular bright field imaging makes use of this property to simultaneously image light and heavy atoms at small collection angles^12^,^13^,^14^. Another new development, segmented detectors^13^, allows for the study of the angular deviation of electron scattering due to microscopic electric fields^15^. Here, we demonstrate the utility of *variable-angle HAADF-STEM* (VA-HAADF) for obtaining three-dimensional (3D) information with true atomic resolution, using the precise determination of dopant atom configurations as an example.

Dynamical diffraction (channeling) of the probe along the atomic columns in HAADF makes the column intensities sensitive to the depth position of an impurity atom^1^,^16^,^17^,^18^,^19^. Quantitative depth position information can be extracted for sufficiently thin samples using quantitative STEM^20^, by comparing the experimental column intensities with calculations for all possible dopant configurations, and determining the most probable dopant position given an experimentally determined noise function^6^,^7^. This method is limited by inherent experimental noise (detector noise, sample instability under the beam, sample contamination, surface amorphous layers, sample imperfections, etc.^21^,^22^), in particular, when intensity differences between different configurations are small. For example, for the Gd dopants in SrTiO_3_ imaged in ref. 6, certain configurations had an uncertainty range that spanned multiple positions, even when the precision is less than a unit cell. Additionally, as will be shown here, certain configurations are prone to generating erroneous position estimates. In principle, one avenue of improving experimental identification of dopant atoms using quantitative STEM lies in reducing the experimental noise, for example, through the development of brighter sources or more stable environments that allow for longer exposures. However, such approaches face inherent limitations, such as beam damage.

Here we show that an alternative route is to utilize the information provided by VA-HAADF. A key feature of VA-HAADF used here is that the angular dependence of the scattering depends on the dopant depth position. A simplified understanding, where we ignore the depth dependent probe intensity oscillation, is shown in Fig. 1(a). As the incident probe channels along a column, atoms deeper in the foil see a more focused probe, resulting in scattering to higher angles (more electrons travelling closer to the nucleus), relative to a dopant atom nearer to the entrance surface. Thus, by selecting certain angular ranges, different dopant atom configurations may be more easily distinguished. In reality, channeling effects are complicated and image simulations must be employed. Figure 1(b) shows multislice calculations of the scattered intensity for different detector angular ranges (in 30 mrad steps) and two different types of dopant atom configurations. For a specific angular range (in this case, 40–100 mrad) the intensity difference between the two configurations is significant. Thus, by combining the depth information from multiple detectors using VA-HAADF, the uncertainty in the dopant depth position measurements will be significantly reduced.

In this paper, we employ simulations and experiments to demonstrate significant improvements in 3D dopant imaging by collecting the HAADF signal within more than one collection angle. We show that one can uniquely identify certain dopant configurations that are indistinguishable in single settings. Furthermore, we demonstrate improvement in the calculated positions to values closer to the correct, discretized positions.

## Results and Discussion

Undoped and Gd-doped SrTiO_3_ were simulated and imaged using two camera lengths that will be referred to hereafter as detector 1 (60–390 mrad) and detector 2 (47–306 mrad). Both detector inner angles are sufficiently high to be adequately described by the incoherent imaging model^23^,^24^,^25^. Figure 2(a) shows experimental and simulated Sr and Ti column intensities (*I*_Sr_ vs. *I*_Ti_) for undoped SrTiO_3_ for thicknesses between three and six unit cells (u.c.) for the two detectors (left and right columns, respectively). Figure 2(b) shows the *I*_Sr_-*I*_Ti_ space for simulated undoped SrTiO_3_ (blue circles) and Gd-doped SrTiO_3_ configurations (yellow rectangular regions). A Gaussian error function, determined from the residual fit of the experimental data in Fig. 2(a) to the simulations, is calculated and overlaid on the 5 u.c. SrTiO_3_ simulation. Such an error function, which takes into account random variability and noise, exists around each point. We point out that other systematic sources of error may exist, however, such as non-uniform surface amorphous areas, errors in the probe and detector calibration, and detector non-uniformity, which may not be captured by the Gaussian error function. While calibration errors can be mostly mitigated through proper fitting to a calibrated data set (i.e. SrTiO_3_), the presence of variable surface layers may non-uniformly alter the intensities of certain columns. While we do not have an estimate of how much variability may exist in these amorphous layers, we point out that we only focus on one particular thickness, which is well-fitted to the calibrated data set, and do not expect the variability in the amorphous layer to be significant compared to the random variability in the measurement.

We focus on the 5 u.c. region, and include Gd-doped SrTiO_3_ simulations of all possible dopant configurations involving single and doubly doped Sr columns (columns containing three or more dopants are highly unlikely for this thickness/concentration^6^). This sub-region is magnified in Fig. 2(c), showing all 15 (5 single, 10 double) configurations for both detectors. The configurations are labeled according to the dopant position along the atomic column, with 1 and 5 marking the top and bottom surface of the sample, respectively, as shown in the inset. From Fig. 2(c), we notice that significant qualitative differences exist between the two detectors for the doubly doped dopant configuration *4, 5*. For detector 1, the *4, 5* configuration is virtually indistinguishable from the *3, 5* case, while these two configurations have a large separation with detector 2. Several other distinguishable/undistinguishable combinations exist; for example, configurations *4*, *5* and *2, 5*. Thus the use of multiple detectors is key to improving the accuracy and precision of quantitative dopant depth measurements.

For quantitative determination of the dopant positions, we can separate the analysis into two steps: determining the number of dopants and determining the location of the dopants. In both steps, the approach is similar: we calculate the probability of each data point being a certain configuration based on the experimentally determined error function; the difference lies in which configurational probabilities contributes to the final probabilities.

### Determining the number of dopants

To determine the number of dopants in a column, we are interested in the most probable configuration among the zero, single, and doubly doped cases. These three are then normalized to give the probabilities of having zero, one, or two dopants in each column. An example calculation is given in the Supplementary Discussion and tabulated in Supplementary Table I. We can calculate this value for both detectors separately, as well as a *combined* probability containing information from both detectors simultaneously, by treating the two as independent measurements. This treatment is valid, if we assume the spread around each discrete configuration is due to random noise. Figure 3 illustrates these probabilities for each configuration and detector, incorporating the experimental error function at each simulated point. The label for each column containing three bars represents a certain dopant configuration (same notation as in Fig. 2). For each detector and configuration, the cumulative probabilities for 0, 1, and 2 dopants are indicated by the color of the bar. From Fig. 3, we see that a doubly doped column in position *2, 3* or *1, 3* would show a nearly even probability split between having 1 or 2 dopants, given the experimental error function. In single doped columns, dopants in position *5* or *4* would show a nearly even probability split if only one detector (1 or 2) is used. Figure 3 shows that while the number of dopant atoms in most configurations can be accurately determined with a single detector setting, certain configurations cannot. However, looking at the probabilities of using the combined detectors in Fig. 3, we see a dramatic improvement in the accuracy of the technique; the probability of measuring the correct number of dopant atoms is greatly increased in each case. Table 1 summarizes the results for the four configurations. While one detector may be better for a certain configuration (such as detector 2 for single dopants in position *5*), using combined probabilities give systematically better results for all configurations. Figure 3 shows that the correct number of dopants can be identified for every configuration when the combined probabilities are used.

### Determining the dopant depth positions

After the number of dopants in a column has been determined, we can determine the dopant positions, by analyzing the subset of probabilities for those configurations (for 1 dopant, we evaluate the probabilities of all 5 possible configurations; for 2 dopants of all 10). Using the measured experimental error function, we calculate the dopant position and uncertainty for a point that lies at each dopant configuration. An example calculation is given in the Supplementary Discussion and tabulated in Supplementary Table II. In Fig. 4, we discuss two cases, a single (Fig. 4a) and double-doped (Fig. 4b) column, respectively. The positions and uncertainties of each configuration are denoted next to the structural model for both individual detectors and the combined detector information. The uncertainties for the two cases using detector 1 are similar to the uncertainties reported in ref. 6. For single dopants, we see that with detector 1, the uncertainty range spans three different atomic positions, with the closest calculated position being incorrect. Detector 2 gives the correct position with sufficient uncertainty to rule out the other nearby positions for this case. In other dopant configurations, the reverse may be true, with detector 1 giving the correct position and/or smaller uncertainties. In Fig. 4b, we see that detector 2 has a smaller uncertainty than detector 1, although both span multiple atomic positions. Furthermore, the closest calculated position using detector 1 is incorrect. However, by combining the probabilities of both detectors together, we see a significant reduction in the uncertainty as well as an accurate measurement, for both configurations.

For a metric of how well each detector performs, we consider: the configurational probability of the *actual* position (higher is better), the average uncertainty (standard deviation of the calculated position), and the difference between the calculated position and the *actual* position (average error). Table 2 sums up these metrics as an *average* overall result across all 15 dopant configurations, grouped by the number of dopants in the column. The improvement column indicates the percent increase from using the combined detector probabilities over the best performing single detector for that metric. Using the information from both detectors gives a substantial improvement across all three metrics.

Measured data points will be located not just at these discrete positions. We simulated the experimental scatter around each dopant configuration in *I*_Sr_ vs. *I*_Ti_ space by incorporating the experimental error function in the data point location. Using 100,000 data points per configuration, we then perform the same calculation for the expected atom position and uncertainty for each of these points. From these calculations, we can track what percent of simulated data points (generated using the experimental error function) around each configuration actually yields calculated positions, or at lies least within the uncertainty range, of the actual position. These two metrics are summarized in the bottom two rows of Table 2 for the individual and combined detector settings, averaged over all 15 dopant configurations. Supplementary Table III lists the individual configuration results. We note that the improvement in the number of simulated data points correctly determined using the combined detector settings is significant (19% for 1 dopant to 14% for 2 dopants). Meanwhile, the data points that lie within the uncertainty range of the actual position show much lower improvement (~3%) using the combined detector information, since the uncertainty range is also being simultaneously reduced. Simultaneously reducing the uncertainty range and average error results in a better likelihood of unambiguously determining a single configuration; meanwhile, the calculation is more often correct. We point out that although we see a simultaneous improvement in both the overall precision and accuracy from using the combined detector data, the values in Table 2 are an average over all configurations, and individual configurations can vary. For instance, as tabulated in Supplementary Table III, the percent of simulated data points correctly identifying the actual dopant position can vary from near perfect (99.9% for the combined detector in the *4, 5* configuration) to undetectable (0% for detector 2 and a dopant in position *1*). These calculations are then particularly valuable, in that they provide a realistic idea of which configurations can be most successfully resolved, and with which detector settings. Once an experimental dopant position is measured and calculated, the Table can be used to determine a confidence level for that calculation.

### Application to experimental HAADF dopant images

Figure 5(a) shows HAADF images from two separate regions in the Gd-doped SrTiO_3_ sample, each taken with the two different detector settings in succession. As discussed above, not every bright Gd-containing column in one setting will result as an equally bright column in the other. The images were taken ~36 s apart, and aligned by eye based on common features (thickness variations, edge features), as some drift occurred between the two recordings. The white triangles/squares in Fig. 5 indicate two Gd-containing columns in each image that we analyze here. Figure 5(b) shows *I*_Sr_ and *I*_Ti_ of the two columns (triangle and square) and the simulated dopant configurations for the two detectors. Select dopant configurations are labeled according to the dopant position(s), while the dashed line indicates a fit to the undoped SrTiO_3_. Qualitatively, while the doped column marked by the triangle might appear to be at position *4* when seen in detector 1, it is clearly much more likely to be at position *3* when taking into account detector 2. Likewise, the dopant marked by the square is probably located at position *4*, when taking the results from both detectors into account. The calculation for the number of dopants and dopant position in each column are given in Table 3. For the doped column in region 1 (square), we see that detector 2 alone would not have been able to distinguish between 1 or 2 dopants, while the combined probability from both detectors is quite convincing, indicating one dopant atom with 78.7% probability. Meanwhile, detector 1 alone would have calculated the incorrect position, while both detectors, when analyzed individually, have an uncertainty that span multiple dopant positions. By using the combined probabilities from both detectors, however, the uncertainty in the measurement is significantly reduced and only one position is identified. The results from the analysis of the column in region 2 (triangle) are similar. Although the final result from both detectors still yields two possible positions, position *2* or *3*), looking at the probabilities of each position indicates that position *3* is the most likely dopant configuration.

## Conclusions

In summary, we have shown that significant improvements in precision *and* accuracy in obtaining three-dimensional dopant configuration information can be realized by using variable-angle information in quantitative HAADF STEM. Using multislice simulations, we showed that one particular detector range is not unilaterally better than the other, but the acquisition of multiple variable angle data and use of compound probabilities will significantly improve dopant depth quantification. We point out that although the dopant position probabilities are conditional probabilities based on the number of estimated dopants in the column, the use of variable angles improves the accuracy and precision of both the number of dopants as well as the their position(s). In general, configurations that are ambiguous in one detector regime can be resolved in the other. For example, within the experimentally determined noise, the probability of determining the correct number of dopants was >63% for all possible sixteen dopant configurations (including zero dopants) when using both detector information, while only 18 out of 32 (56%) configurations met that requirement for a single detector. Likewise, the calculated position is ~17% more likely to be the actual position, as calculated positions from a single detector can sometimes be erroneous.

**Figure 1:**
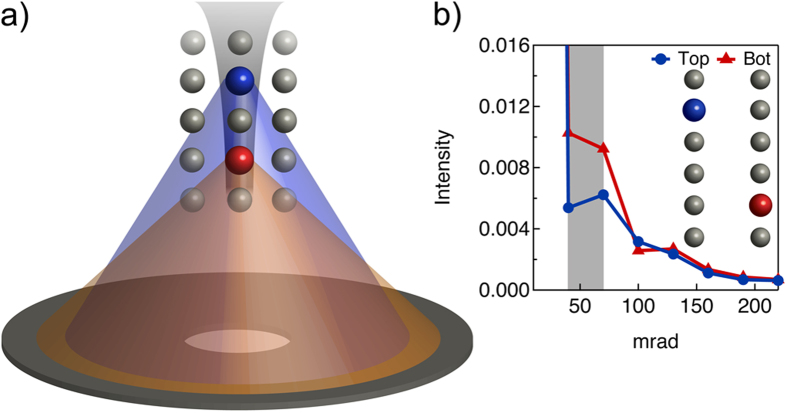
*Principle of VA-HAADF to accurately determine dopant depth positions*. (**a**) Schematic showing how beam channeling along an atomic column can result in angle-dependent scattering in STEM. Atoms further down a column “see” a more focused probe and consequently scatter more to higher angles. (**b**) Simulated multislice results showing scattered intensity as a function of annular detector angle (in 30 mrad segments, as exemplified by the gray box).

**Figure 2:**
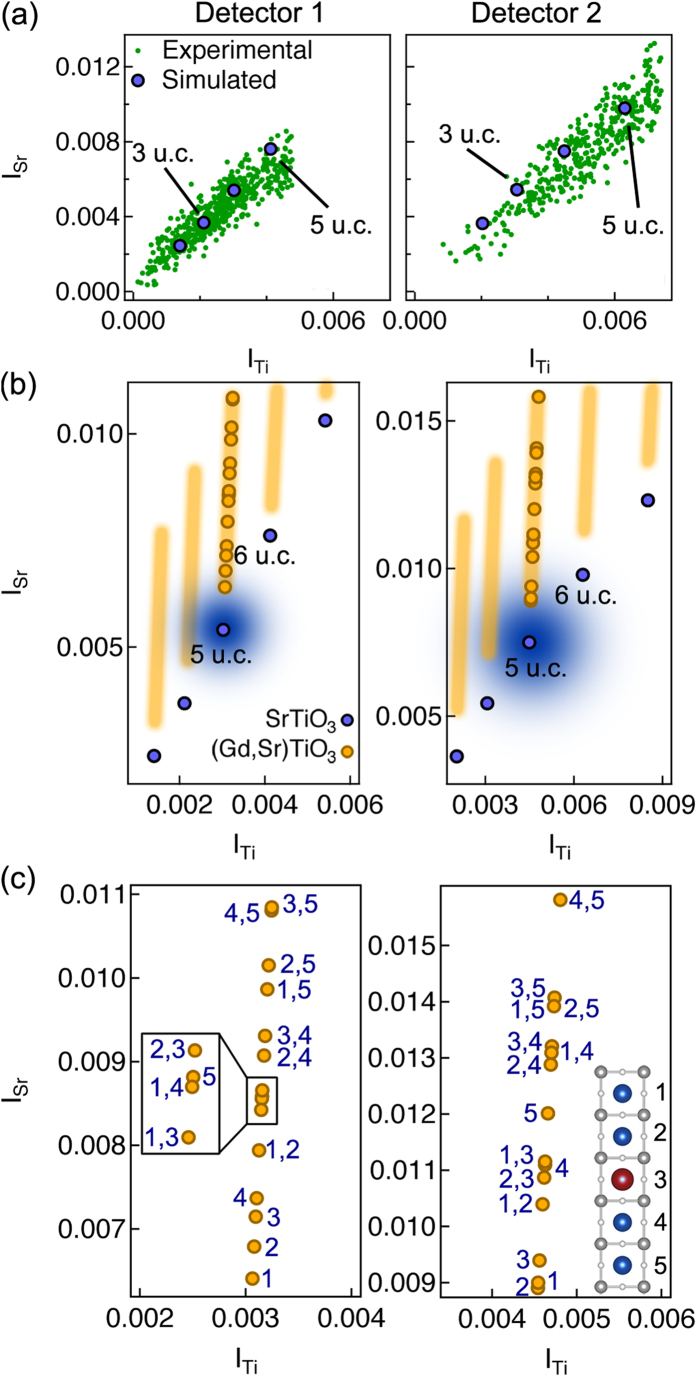
*Experimental and simulated column intensities of undoped and Gd-doped SrTiO*_*3*_ *for the two detectors*. (**a**) Experimental and simulated *I*_Sr_ vs. *I*_Ti_ for the undoped SrTiO_3_ of 3 to 6 u.c. thicknesses for detector 1 (left) and detector 2 (right). Experimental data points are after subtraction of a constant background, *I*_B_ = 0.003. (**b**) *I*_Sr_ vs. *I*_Ti_ for simulated undoped and Gd-doped SrTiO_3_ for detector 1 (left) and detector 2 (right). Individual configurations for 5 u.c. thick samples are shown for the Gd-doped SrTiO_3_ while rectangular bars mark the general areas in other regions where doped configurations would appear. A 2D Gaussian, with standard deviation calculated from the residual of the data in (**a**), is superimposed on the undoped 5 u.c. position. (**c**) Magnified region of simulated *I*_Sr_ vs. *I*_Ti_ for 5 unit cell thickness for detector 1 (left) and detector 2 (right). Individual dopant configurations are labeled according to the position(s) of the dopant(s). The dopant positions are shown in the inset.

**Figure 3:**
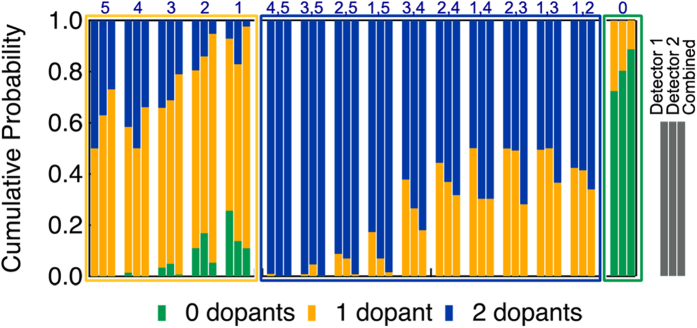
*Cumulative probabilities for detecting the number of dopants in a column*. Probabilities of detecting 0, 1 or 2 dopants in each dopant configuration, given the experimental error function determined from Fig. 2. Each group of three bars represents (from left to right) the probabilities for detector 1, detector 2, and combining the information from both detectors.

**Figure 4:**
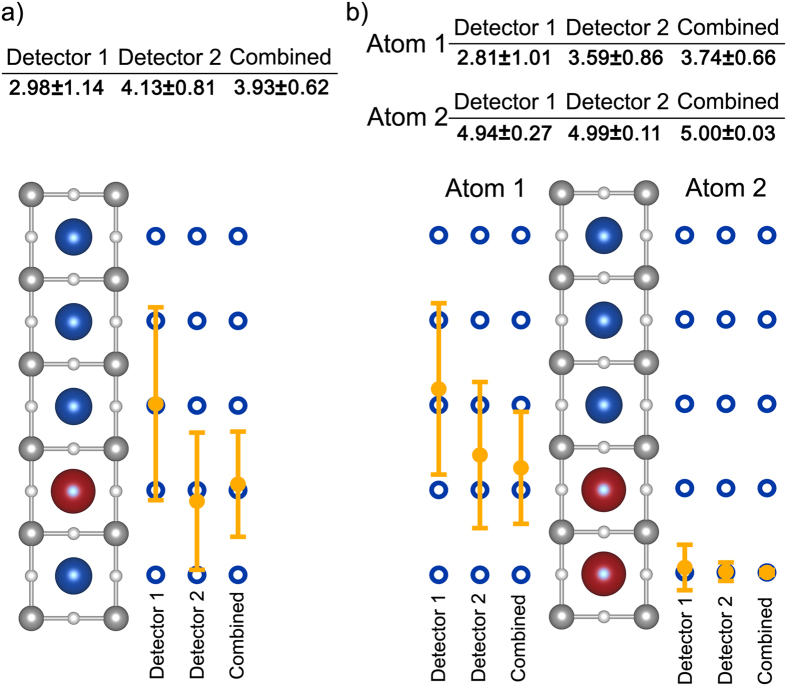
*Single detector vs. combined detector information about dopant depth positions*. Calculated positions and uncertainties for (**a**) a single dopant located at position 4 and (**b**) two dopants located at position 4 and 5. From left to right, filled yellow points and error bars represent the position and uncertainty of detector 1, detector 2, and the combined detector information, respectively. For (**b**), both atom positions and uncertainties are given and plotted separately.

**Figure 5:**
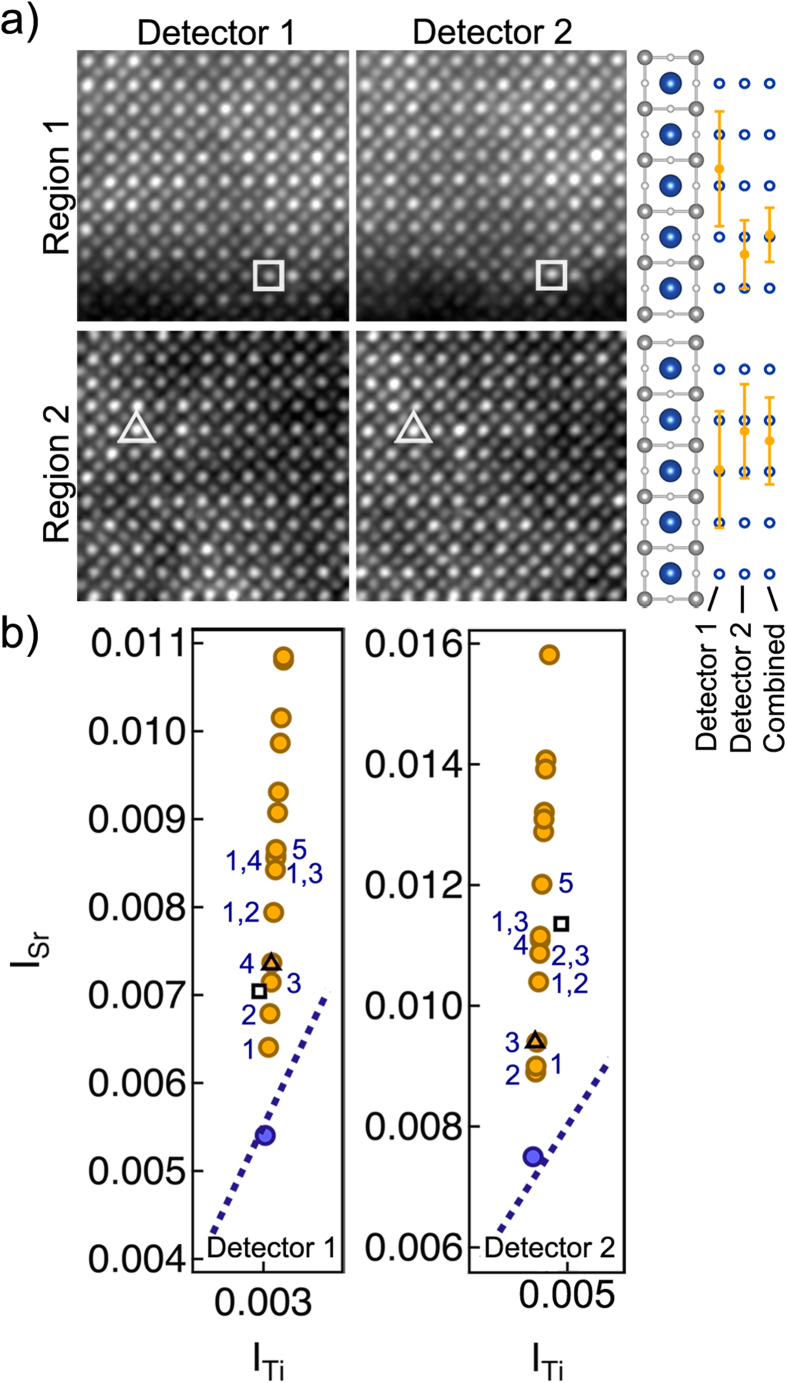
*Experimental analysis of Gd-doped SrTiO*_*3*_. (a) HAADF STEM images of two regions containing dopant atoms in Gd-doped SrTiO_3_ for detector 1 (left) and detector 2 (right). White squares/triangles indicate the doped column of interest. A low band pass filter was applied to the images. The quantitative analysis was performed on the unfiltered, raw data. (**b**) Simulated *I*_Sr_ vs. *I*_Ti_ dopant configurations compared to experimental values shown in (**a**). Selected dopant configurations are labeled for each detector.

While the present study utilized only two angular regimes due to practical constraints, it is sufficient to provide proof of the usefulness of VA-HAADF. The method can and should be extended to include additional angular ranges, and is restricted only by detector limitations and, of course, to remain with in the HAADF imaging mode. As seen in Fig. 1(b), certain angular ranges can have dramatic differences in the scattered intensities of a specific configuration. Furthermore, parallel data acquisition would allow for minimizing drift between acquisitions that makes locating the same area difficult in the absence of discernable features such as edges, and to avoid the beam damage caused by multiple exposures. To that end, the development and implementation of segmented^13^,^26^,^27^,^28^ or pixelated HAADF detectors^29^,^30^ is paramount and will make it easier to implement VA-HAADF for any number of detectors and angular ranges. It is important, however, that these new generation of detectors have response characteristics that meets the requirements for quantitative HAADF-STEM^31^. Furthermore, the complementary information acquirable through VA-HAADF will likely be useful not only for dopant depth identification, but serve to generally improve STEM image contrast and its interpretability for the analysis of strain, defects, and enhanced structural identification.

## Methods

SrTiO_3_ films containing a Gd dopant concentration of 4 at% were grown by hybrid molecular beam epitaxy^32^. An undoped SrTiO_3_ single crystal was used as a calibration sample to determine the experimental noise, closely following the procedure described elsewhere^6^. Plan-view TEM samples were prepared by mechanical polishing using a 1° wedge angle. HAADF STEM images were recorded on a 300 kV FEI Titan (C_s_ = 1.2 mm) with a 9.6 mrad convergence angle. Images were 512 × 512 pixels at 50 μs dwell times, with roughly 160 atom columns per image. Two separate camera lengths, 100 and 130 mm, were used in succession, and referred to above as detector 1 and detector 2 settings. The inner angle of each camera length was measured separately, and the outer angle calculated from the detector image (60–390, 47–306 mrad for 100 and 130 mm camera lengths, respectively)^31^. The contrast of the undoped columns is similar with both angular regimes^33^. Thus changes in the intensity ratios of the Sr/Ti-O columns can be attributed to the Gd dopants in the Sr columns. The projector lens system of each camera length was calibrated and aligned before each acquisition, as described in ref. 31. Frozen phonon simulations were carried out using the Kirkland program suite^34^ for all possible dopant configurations involving one or two Gd dopants. Simulated structures were all 5 u.c. thick and did not include the possibilities of Sr adatoms. The frozen phonon simulations used a 1024 × 1024 pixel mesh for a 15.62 × 15.62 Å supercell. The sample thickness was limited to below 4 nm to avoid the probe intensity oscillations due to channeling along a zone axis that would prohibit unambiguous identification of dopant atom depth (*z*) positions^6^. To reduce computation times, simulations included TDS effects by using a calibrated intensity ratio, as described in ref. 6.

Image intensities were normalized to the incident probe intensity, as discussed in ref. 20, separately for each detector configuration. To account for the detector non-uniformity, the incident probe intensity on the detector was obtained by averaging the signal between 60–120 mrad and 47–141 mrad for the two detectors^35^. Atomic column intensities (*I*_Sr_ and *I*_Ti_ for Sr and Ti-O columns, respectively) were extracted by averaging the intensity around each atomic column centroid with a radius ¼ the length of the unit cell (~10 pixels). These integrated intensities are less sensitive to defocus and source coherence, and allow for more robust comparisons to simulations^36^. As noted in ref. 6, a substantial portion of the signal arises from amorphous surface contributions in such extremely thin samples (~2 nm). A constant background, *I*_B_ = 0.003, due to the surface contribution, was subtracted from the experimental data points for comparison with simulations.

## Additional Information

**How to cite this article**: Zhang, J. Y. *et al.* Variable-angle high-angle annular dark-field imaging: application to three-dimensional dopant atom profiling. *Sci. Rep.* **5**, 12419; doi: 10.1038/srep12419 (2015).

## Supplementary Material

Supplementary Information

## Figures and Tables

**Table 1:** Probability of determining the correct number of dopants.

Dopant Position(s)	Probability of correct number of dopants			Improvement
	Detector 1	Detector 2	Combined	
5	50.0%	62.9%	73.1%	16%
4	57.0%	50.1%	66.1%	16%
2,3	50.1%	50.1%	71.9%	44%
1,3	50.6%	50.0%	63.5%	25%

Probabilities of determining the correct number of dopants for select dopant configurations, using detector 1, detector 2, and the combined information from both detectors. The improvement is the percent increase of the combined detector over the best performing single detector.

**Table 2:** Performance comparisons of individual and combined detectors.

	1 Dopant				2 Dopants			
	Detector 1	Detector 2	Combined	Improvement	Detector 1	Detector 2	Combined	Improvement
Config. Prob	38.2%	45.1%	57.4%	27%	22.8%	31.4%	44.6%	42%
Uncertainty	1.06	0.78	0.68	13%	0.73	0.61	0.54	11%
Avg. Error	0.67	0.48	0.35	27%	0.47	0.44	0.33	25%
% Correct	35.7%	44.3%	52.6%	19%	51.0%	55.6%	63.5%	14%
%In Range	67.8%	58.4%	70.3%	4%	69.5%	62.7%	71.1%	2%

Performance metrics for detector 1, detector 2, the combined detectors, and the improvement of the combined detector over the best performing single detector, grouped by number of dopants in the column. Row 1 indicates the configurational probability of the actual dopant position. Row 2 indicates the uncertainty range (standard deviation) of the calculated position. Row 3 indicates the distance between the calculated position and the actual position. Row 4 indicates the percent of data points (out of 100,000 simulated points) that, when rounded to the nearest position, are correctly identified. Row 5 indicates the percent of data points that are within the uncertainty range of the calculated position.

**Table 3:** Summary of probabilities for experimental dopant number and positions.

# Dopants	Detector 1	Detector 2	Combined	Detector 1		Detector 2		Combined	
				Position	Probability	Position	Probability	Position	Probability
0	5.1%	0%	0%	5	0.0279	5	0.4179	5	0.0742
1	64.9%	49.5%	78.7%	4	0.2491	4	0.5311	4	0.8429
2	30.1%	50.5%	21.3%	3	0.2734	3	0.0340	3	0.0592
				2	0.2607	2	0.0071	2	0.0118
				1	0.1889	1	0.0099	1	0.0119
**Region 1**				**2.67** **±** **1.12**		**4.34** **±** **0.67**		**3.96** **±** **0.53**	
0	1.5%	4.8%	0.2%	5	0.0692	5	0.0026	5	0.0008
1	57.3%	63.8%	73.1%	4	0.2978	4	0.0441	4	0.0605
2	41.1%	31.3%	26.8%	3	0.2861	3	0.3489	3	0.4599
				2	0.2202	2	0.2929	2	0.2970
				1	0.1267	1	0.3115	1	0.1818
**Region 2**				**2.96** **±** **1.14**		**2.21** **±** **0.92**		**2.40** **±** **0.85**	

Calculated probabilities for the number and location of dopants for the two atomic columns marked by squares (region 1) and triangles (region 2) in Fig. 4.
