# Supplementary material for: Variable-angle high-angle annular dark-field imaging: application to three-dimensional dopant atom profiling
Source: Sci Rep. 2015 Jul 24;5:12419. doi: 10.1038/srep12419 (PMC4513304; doi:10.1038/srep12419)
Supplement: Supplementary Information [file srep12419-s1.pdf]

Supplementary Material:

Variable-angle high-angle annular dark-field imaging: application to three-dimensional dopant atom profiling

Jack Y. Zhang, Jinwoo Hwang, Brandon J. Isaac, and Susanne Stemmer
Materials Department, University of California, Santa Barbara, California 93106-5050, U. S. A.

Supplementary Discussion

An example calculation for the atomic column in region 1 from Fig. 5 and Table III are given as follows. The probability of an experimental data point corresponding to a calculated configuration is given by:

$$p_i = \frac{norm_i(t)}{\sum_n norm_n(t)}, \quad \text{(S1)}$$

where t indicates the distance in I_{Sr} vs. I_{Ti} space between the experimental and simulated point, normalized to the standard deviation of the Gaussian error function, and $norm_i(t)$ represents the value of the Gaussian probability distribution function for a given distance t. The experimental Gaussian error function is modeled as a normal distribution with mean at the simulated intensity value for each configuration, and standard deviation determined experimentally from the calibration sample (Fig. 2). The subscript i represents an experimental point while n represents each dopant configuration considered. The expectation value for a dopant position is given as:

$$\mu = \sum_i z_i p_i \,, \quad \text{(S2)}$$

where z represents the atom position, with uncertainty:

$$\sigma = \sqrt{\sum_i p_i \left(z_i - \mu\right)^2} \quad \text{(S3)}$$

Supplementary Table I lists the dopant number calculations for the atom in region 1 using detector 1 (Fig. 5). The distance t is calculated between the experimental point and each simulated configuration, normalized by the standard deviation. The values for $norm_i(t)$ can be found using any statistical software. $\sum norm_c(t)$ is calculated separately for the 0,

1, and 2 dopant cases. Most probabilistic values of $norm_c(t)$ for 0, 1, or 2 dopants are listed in the next column. Values for p_i are calculated according to Eq. S1, using the corresponding $\sum norm_c(t)$. The probabilities of having 0, 1, or 2 dopants in the column are calculated with Eq. S1 using values only from the most probabilistic column.

Supplementary Table I. *Dopant number calculations for detector 1.* Values for calculating the individual configurational p_i and the probabilities of having 0, 1, or 2 dopants in the square column from Fig. 5.

	I_{Sr}	I_{Ti}	t	norm (t)	Σnorm(t)	most prob.	p_i	prob. config.
Experimental Position	0.00704	0.00295						
Simulated Positions								
5	0.00859	0.00315	2.151	0.0395			0.0279	
4	0.00737	0.00310	0.496	0.353			0.249	
3	0.00715	0.00309	0.245	0.387			0.273	
2	0.00679	0.00308	0.394	0.369			0.261	
1	0.00641	0.00306	0.894	0.267	1.416	0.387	0.189	0.649
4,5	0.0108	0.00324	5.228	4.64E-7			1.43E-6	
3,5	0.0108	0.00325	5.279	3.55E-7			1.10E-6	
2,5	0.0102	0.00322	4.320	3.53E-5			1.09E-4	
1,5	0.00986	0.00320	3.923	1.81E-4			5.60E-4	
3,4	0.00931	0.00318	3.154	0.00276			0.00852	
2,4	0.00907	0.00317	2.830	0.00727			0.0225	
1,4	0.00856	0.00315	2.120	0.0422			0.130	
2,3	0.00866	0.00316	2.254	0.0314			0.0972	
1,3	0.00842	0.00315	1.934	0.0614			0.190	
1,2	0.00794	0.00313	1.269	0.178	0.324	0.178	0.551	0.301
0	0.00540	0.00302	2.266	0.0306	0.0306	0.0306	1	0.0507

The calculation for the position of the dopant is given in Supplementary Table II. The expected value is given as the sum of $z_i p_i$ while the uncertainty is the square root of the sum of the third column. The calculation for detector 2 is done the same way, while for the combined detector, respective values of $norm_c(t)$ for both detectors are multiplied together, and all subsequent calculations remain the same.

Supplementary Table II. *Dopant position calculation for detector 1*. Calculation for the position of the dopant, from the atom column marked by squares in Fig. 5.

Atom Positions	z_i*p_i	$(z_i-\mu)^2$*p_i
5	0.138	0.150
4	0.994	0.444
3	0.819	0.0310
2	0.522	0.115
1	0.190	0.525
Sum	2.66	1.27

Supplementary Table III. *Expected positions and accuracy assessment for all dopant configurations*. Calculation results from 100,000 randomly scattered simulated points around each dopant configuration, grouped by number of dopants in the atomic column. Results are given for each individual detector as well as the combined setting. For each configuration: row 1 is the average calculated position and uncertainty for all data points around that configuration; row 2 is the percent of points with calculated positions that round to the actual dopant position; row 3 is the percent of points with calculated positions that lie within the uncertainty range of the actual dopant position.

Pos.		Detector 1	Detector 2	Combined	Pos.		Detector 1		Detector 2		Combined	
							Atom 1	Atom 2	Atom 1	Atom 2	Atom 1	Atom 2
	Exp. Val.	4.30±0.78	4.50±0.56	4.74±0.35		Exp. Val.	2.76±0.93	4.83±0.32	3.37±0.75	4.95±0.15	3.48±0.64	4.99±0.06
5	% Correct	50.9%	65.8%	81.8%	4,5	% Correct	0%	90.3%	54.7%	98.7%	62.2%	99.9%
	In Range	75.5%	80.1%	84.9%		In Range	21.7%	95.5%	76.8%	99.7%	79.6%	99.9%
	Exp. Val.	3.08±1.08	3.89±0.81	3.79±0.69		Exp. Val.	2.78±0.92	4.84±0.31	2.23±0.91	4.63±0.43	2.56±0.80	4.90±0.22
4	% Correct	22.2%	48.0%	58.2%	3,5	% Correct	73.3%	91.3%	14.0%	74.5%	44.2%	95.8%
	In Range	54.2%	65.8%	72.7%		In Range	93.2%	96.0%	43.3%	75.8%	73.1%	96.1%
	Exp. Val.	2.86±1.09	2.36±0.93	2.46±0.84		Exp. Val.	2.37±0.93	4.56±0.52	2.17±0.89	4.59±0.45	2.23±0.84	4.73±0.34
3	% Correct	48.3%	19.3%	30.2%	2,5	% Correct	62.3%	65.3%	87.6%	68.2%	81.3%	80.7%
	In Range	84.3%	50.0%	58.6%		In Range	88.6%	78.7%	95.9%	69.6%	92.1%	81.4%
	Exp. Val.	2.53±1.07	2.10±0.86	2.08±0.79		Exp. Val.	2.21±0.91	4.40±0.60	2.17±0.89	4.58±0.45	2.13±0.88	4.65±0.38
2	% Correct	51.0%	88.5%	80.3%	1,5	% Correct	1.9%	49.8%	0.2%	68.1%	0.4%	70.9%
	In Range	84.3%	96.2%	92.7%		In Range	11.5%	65.5%	1.6%	69.5%	11.8%	71.5%
	Exp. Val.	2.23±1.02	2.14±0.87	1.94±0.79		Exp. Val.	1.92±0.82	4.01±0.71	1.97±0.82	4.30±0.53	1.91±0.78	4.25±0.45
1	% Correct	6.0%	0%	12.5%	3,4	% Correct	6.8%	59.2%	2.0%	58.7%	1.0%	67.4%
	In Range	40.9%	0%	42.5%		In Range	26.2%	77.3%	11.7%	62.2%	5.5%	69.1%
						Exp. Val.	1.81±0.78	3.84±0.74	1.89±0.79	4.13±0.56	1.83±0.75	4.05±0.46
					2,4	% Correct	80.0%	57.2%	93.5%	65.5%	90.1%	74.3%
						In Range	90.8%	79.0%	96.5%	70.3%	95.0%	77.2%
						Exp. Val.	1.60±0.68	3.48±0.78	1.94±0.81	4.24±0.54	1.69±0.73	3.98±0.43
					1,4	% Correct	39.7%	40.2%	3.3%	61.6%	23.3%	82.1%
						In Range	73.0%	67.0%	12.4%	65.5%	61.2%	85.0%
						Exp. Val.	1.64±0.70	3.55±0.77	1.43±0.54	2.87±0.56	1.46±0.52	2.92±0.50
					2,3	% Correct	64.5%	50.7%	20.5%	77.6%	38.1%	78.5%
						In Range	77.9%	71.3%	28.4%	81.2%	43.1%	81.1%
						Exp. Val.	1.55±0.65	3.39±0.78	1.48±0.57	3.02±0.57	1.44±0.53	2.98±0.52
					1,3	% Correct	47.1%	62.5%	68.5%	75.5%	67.3%	74.0%
						In Range	79.0%	81.1%	87.6%	80.7%	79.7%	77.3%
						Exp. Val.	1.39±0.56	3.10±0.78	1.36±0.50	2.68±0.53	1.27±0.42	2.57±0.48
					1,2	% Correct	72.4%	5.2%	91.8%	27.4%	92.3%	45.8%
						In Range	93.0%	23.1%	98.1%	28.0%	94.9%	47.6%
